# A benchmarking framework for perturbational signature recontextualization across biological contexts

**DOI:** 10.64898/2026.08.14.744937

**Published:** 2026-10-22

**Authors:** Andrew Chen, Noa Rappaport, Nick Schork, Thomas Girke, Stefano Monti

## Abstract

Perturbational transcriptomics is a powerful tool for understanding gene function and drug effects, yet predicting how perturbations manifest across different biological contexts remains a central challenge, limiting translation from model systems to clinically relevant tissues. Despite growing interest in this problem, benchmarking efforts have been hindered by inconsistent evaluation tasks, heterogeneous metrics, and limited assessment across perturbation types and biological systems. Here, we introduce a benchmarking framework for cross-context perturbation-signature prediction (a task we define as *signature recontextualization* ), grounded in explicit definitions of the prediction task, target-data availability, and evaluation metrics centered on signature recovery. The framework evaluates prediction performance across three target-context data regimes: (1) control only, where only control profiles from the target context are measured; (2) low coverage, where a limited subset of perturbations in the target context are measured; and (3) high coverage, where most perturbations in the target context are measured. This design enables systematic assessment of how prediction performance depends on target-context sample size while providing a standardized basis for comparing methods. We evaluate newly developed projection-based (projectCor) and network-based (netProp) methods alongside deep learning-based foundation models ( *sc* GPT, STACK) and statistical baselines. The benchmark spans diverse perturbational datasets: CRISPR knockdowns and drug perturbations in cell lines, plus in vivo chemical perturbations in rat tissues from DrugMatrix, extending evaluation beyond isolated cell-line models to tissue-level responses. For each method, evaluation was limited to the datasets, and perturbation types each model supports. Across tasks, projection and network propagation approaches show strong flexibility across perturbation types and biological contexts, and in several cases match or exceed the performance of deep learning and foundation models. We further show that perturbation predictability varies substantially with pathway conservation, transcriptional response strength, and baseline similarity between source and target contexts. All datasets, methods, and evaluation utilities are released as an open-source R package ( *sigRecon* ), providing a foundation for reproducible benchmarking and future method development.

